# Assessment of Dynamic Changes in Stressed Volume and Venous Return during Hyperdynamic Septic Shock

**DOI:** 10.3390/jpm12050724

**Published:** 2022-04-29

**Authors:** Athanasios Chalkias, Eleni Laou, Nikolaos Papagiannakis, Vaios Spyropoulos, Evaggelia Kouskouni, Kassiani Theodoraki, Theodoros Xanthos

**Affiliations:** 1 Department of Anesthesiology, Faculty of Medicine, University of Thessaly, 41500 Larisa, Greece; elenilaou1@gmail.com; 2 Outcomes Research Consortium, Cleveland, OH 44195, USA; 3 Hellenic Society of Cardiopulmonary Resuscitation, 11528 Athens, Greece; 4 First Department of Neurology, Eginition University Hospital, Medical School, National and Kapodistrian University of Athens, 11528 Athens, Greece; nikolas.papagia@gmail.com; 5 Medical Supervision S.A., 10680 Athens, Greece; sp.vaios@gmail.com; 6 Department of Biopathology, Aretaieion University Hospital, Medical School, National and Kapodistrian University of Athens, 11528 Athens, Greece; ekouskouni@yahoo.com; 7 Department of Anesthesiology, Aretaieion University Hospital, National and Kapodistrian University of Athens, 15772 Athens, Greece; ktheodoraki@hotmail.com; 8 School of Medicine, European University Cyprus, Nicosia 2404, Cyprus; theodorosxanthos@yahoo.com

**Keywords:** septic shock, venous return, mean circulatory filling pressure, stressed volume, unstressed volume, rest volume, cardiovascular dynamics, hemodynamics, anesthesiology, intensive care medicine

## Abstract

The present work investigated the dynamic changes in stressed volume (*Vs*) and other determinants of venous return using a porcine model of hyperdynamic septic shock. Septicemia was induced in 10 anesthetized swine, and fluid challenges were started after the diagnosis of sepsis-induced arterial hypotension and/or tissue hypoperfusion. Norepinephrine infusion targeting a mean arterial pressure (MAP) of 65 mmHg was started after three consecutive fluid challenges. After septic shock was confirmed, norepinephrine infusion was discontinued, and the animals were left untreated until cardiac arrest occurred. Baseline *Vs* decreased by 7% for each mmHg decrease in MAP during progression of septic shock. Mean circulatory filling pressure (Pmcf) analogue (Pmca), right atrial pressure, resistance to venous return, and efficiency of the heart decreased with time (*p* < 0.001 for all). Fluid challenges did not improve hemodynamics, but noradrenaline increased *Vs* from 107 mL to 257 mL (140%) and MAP from 45 mmHg to 66 mmHg (47%). Baseline Pmca and post-cardiac arrest Pmcf did not differ significantly (14.3 ± 1.23 mmHg vs. 14.75 ± 1.5 mmHg, *p* = 0.24), but the difference between pre-arrest Pmca and post-cardiac arrest Pmcf was statistically significant (9.5 ± 0.57 mmHg vs. 14.75 ± 1.5 mmHg, *p* < 0.001). In conclusion, the baseline *Vs* decreased by 7% for each mmHg decrease in MAP during progression of hyperdynamic septic shock. Significant changes were also observed in other determinants of venous return. A new physiological intravascular volume existing at zero transmural distending pressure was identified, termed as the rest volume (*Vr*).

## 1. Introduction

The traditional management of shock focuses on the regulation of left ventricular cardiac output (CO). However, it is the venous return theory that provides an understanding of the circulation, emphasizing that CO is associated with, and regulated by, the amount of blood returning to the heart. In general, venous return occurs because of a pressure gradient between the periphery and the right atrium. As a matter of fact, not all the blood leaving the venous system returns to the heart at the same time because the largest quantity remains within the veins to regulate venous return [1]; therefore, approximately 30% of the total blood volume (TBV) represents stressed volume (*Vs*, i.e., the volume in blood vessels when transmural distending pressure (Ptm) is above zero), while the remaining 70% is unstressed volume (*Vu*), i.e., the volume in blood vessels when Ptm equals zero.

The modification of the venous system that occurs in sepsis is poorly understood. Experimental studies have indicated a diverse pathophysiology with biphasic hemodynamic responses and/or hyperdynamic hypotensive circulatory states [2,3,4], suggesting a disproportionate impairment in peripheral vasoregulation [5]. Sepsis increases venous capacitance and decreases systemic vascular resistance (SVR), leading to cardiovascular compromise and tissue hypoperfusion. In septic shock, the TBV status is unchanged, but the progressive vasodilation shifts a portion of the *Vs* to *Vu*, which decreases mean circulatory filling pressure (Pmcf) and venous return [6]. 

The use of the *Vs*:*Vu* ratio introduced novel strategies for fluid resuscitation and vasopressor administration. Nevertheless, the current recommendations on sepsis and septic shock have failed to reach hemodynamic goals [7]. After decades of research, it seems that the optimal management requires a basic understanding of the underlying evolving pathophysiology and an individualized, physiology-guided strategy [8]. An important asset to this would be the comprehension of *Vs*:*Vu* ratio changes during progression of the condition. In the present study, we aimed to elucidate this topic in greater detail. To this end, we investigated the dynamic changes in *Vs* and other determinants of venous return during progression and resuscitation of hyperdynamic septic shock in an experimental swine model.

## 2. Materials and Methods

### 2.1. Extrapolation Model of Calculation of Stressed Volume 

An extrapolation model was created to assess circulatory volumes in steady-state and pathophysiological conditions using 20-kg Landrace–Large White swine. As the animals’ baseline hemodynamics closely resemble human hemodynamics [9,10], we accepted that 30% of their TBV represents *Vs* and the remaining 70% is *Vu* [11,12,13,14]. The TBV of the Landrace–Large White swine is 7% of the total body weight, i.e., 1400 mL for a 20 kg animal, and therefore, their baseline *Vs* is 420 mL.

We have recently shown that the blood volume that has to be removed from the 20-kg swine to induce cardiac arrest is ≈860 mL [15]. This volume includes the *Vs* and the *Vu* that converts to *Vs* during hemorrhage [14,16]. Considering that the *Vs* is 420 mL, the blood volume mobilized from the splanchnic and other compliant veins to maintain Ptm > 0, and thus *Vs* and venous return, in the 20-kg swine during hemorrhage is 440 mL [15]. Although in severe hypovolemia the homeostatic mechanisms involved in hemodynamics and CO regulation may empty the splanchnic reservoir [17], the remaining 540 mL of the 1400 mL of blood in our animals was volume that was not mobilized from the venous pool, probably due to depletion of sympathoadrenal system reserves or splanchnic sequestration, or mobilization could have occurred only with the use of exogenous vasopressor. This volume can be characterized as the “rest volume” (*Vr*), i.e., the volume that cannot be mobilized without the use of an external vasopressor or without decreasing arterial and/or venous resistance. The *Vs* and the *Vu* (i.e., the volume that can be converted to *Vs* or *Vr*) constitute the potential total circulating blood volume (*Vc*). In our model, the following apply:

(1) Total blood volume (mL)=Vc+Vr=(Vs+Vu)+Vr

and

Steady state: *Vs* = 420 mL, *Vu* = 440 mL, *Vr* = 540 mL (TBV = 1400 mL).

During hemorrhage: *Vs* = 420 mL + 440 mL from *Vu* (to maintain Ptm > 0), *Vr* = 540 mL.

Hypovolemic cardiac arrest: 860 mL removed and *Vr* = 540 mL (Ptm ≈ 0).

In summary, in the hemorrhagic model, the *Vs* (420 mL in the 20-kg swine with TBV 1400 mL) was related to a MAP of 88.4 mmHg, while the *Vr* (540 mL) was the blood volume at MAP 30 mmHg (cardiac arrest) [15]. The *Vs*, *Vu*, and *Vr* during hemorrhage are depicted in Figure S1. The extrapolation of the aforementioned baseline data from the hemorrhagic model to animals of the same age, weight, TBV, and baseline hemodynamics [9,15] allows the study of circulatory volumes in other experimental conditions using linear regression analysis.

### 2.2. Experimental Model

#### 2.2.1. Ethics Approval

Taking into consideration the principles of 3R, i.e., Replacement, Reduction, and Refinement, which represent a responsible approach for performing more humane animal research [18], we conducted a post hoc analysis of high-quality hemodynamic data derived from a previous study investigating resuscitation in hyperdynamic septic shock [9]. The original protocol was approved by the General Directorate of Veterinary Services (license No. 26, 10 January 2012) according to the national legislation regarding ethical and experimental procedures. These procedures conformed to the guidelines from Directive 2010/63/EU of the European Parliament on the protection of animals used for scientific purposes or the current National Institutes of Health guidelines. The manuscript adheres to the applicable ARRIVE 2.0 and Minimum Quality Threshold in Pre-Clinical Sepsis Studies (MQTiPSS) guidelines [19,20].

#### 2.2.2. Study Objectives

The primary objective was to assess the dynamic changes in *Vs* and other determinants of venous return during progression and resuscitation of hyperdynamic septic shock. Secondary objective was to measure Pmcf after sepsis-induced cardiac arrest.

#### 2.2.3. Origin and Source of the Animals

This analysis included 10 healthy female Landrace–Large White piglets aged 19–21 weeks with average weight of 20 ± 1 kg, all purchased from the same breeder (Validakis, Koropi, Greece). One week prior to the experiments, the animals were transported to the research facility (Experimental-Research Center Elpen, European Ref Number EL 09 BIO 03) and were acclimatized to laboratory conditions, as previously described [10]. The day before the experimentation, the animals were fasted, but access to water was ad libitum. All animals received anesthetic and surgical procedures in compliance with the Guide for the Care and Use of Laboratory Animals [21].

#### 2.2.4. Animal Preparation

The animals were premedicated with intramuscular ketamine hydrochloride (Merial, Lyon, France), 10 mg·kg^−1^, midazolam (Roche, Athens, Greece), 0.5 mg·kg^−1^, and atropine sulphate (Demo, Athens, Greece), 0.05 mg·kg^−1^, and were subsequently transported to the operation research facility. Intravascular access was obtained through the auricular veins, and induction of anesthesia was achieved with an intravenous bolus dose of propofol (Diprivan 1% *w*/*v*; AstraZeneca, Luton, United Kingdom), 2 mg·kg^−1^, and fentanyl (Janssen Pharmaceutica, Beerse, Belgium), 2 μg·kg^−1^. While breathing spontaneously, the animals were intubated with a size 6.0 mm cuffed endotracheal tube, which was secured on the lower jaw. Successful intubation was ascertained by auscultation of both lungs while ventilated with a self-inflating bag. 

The animals were then immobilized in the supine position on the operating table and were volume-controlled ventilated (tidal volume 10 mg·kg^−1^, inspiratory-to-expiratory time ratio 1:2, positive end-expiratory pressure 0 cm H_2_0, fraction of inspired oxygen 0.21; Siare Alpha-Delta Lung Ventilator; Siare s.r.l. Hospital Supplies, Bologna, Italy) [22]. Additional amounts of 1 mg·kg^−1^ propofol, 0.15 mg·kg^−1^ cis-atracurium, and 4 μg·kg^−1^ fentanyl were administered intravenously to ascertain synchrony with the ventilator. Amounts of propofol 0.1 mg·kg^−1^·min^−1^, cis-atracurium 20 μg·kg^−1^·min^−1^, and fentanyl 0.6 μg·kg^−1^·min^−1^ were administered to maintain adequate anesthetic depth, assessed by the jaw tone, throughout the study [9,10,22]. Normocapnia was achieved using continuous monitoring of end-tidal carbon dioxide (ETCO_2_, Tonocap TC-200-22-01; Engstrom Division, Instrumentarium Corp, Helsinki, Finland), and the respiratory rate was adjusted to maintain ETCO_2_ 35–40 mmHg. Pulse oximetry was monitored throughout the experiment. Body temperature was monitored by a rectal temperature probe and was maintained between 38.5 °C and 39.5 °C with a heating blanket [22].

Electrocardiographic monitoring was used using leads I, II, III, aVR, aVL, and aVF, which were connected to a monitor (Mennen Medical, Envoy; Papapostolou, Athens, Greece) that electronically calculated the heart rate. For measurement of the aortic pressures, an arterial catheter (model 6523, USCI CR, Bart; Papapostolou, Athens, Greece) was inserted and moved forward into the descending aorta after surgical preparation of the right internal carotid artery. A FloTrac sensor kit was connected to the arterial line and coupled to a Vigileo monitor (FloTrac/Vigileo; Edwards Lifescience, Irvine, CA, USA). Then, the internal jugular vein was cannulated, and a Swan–Ganz catheter (Opticath 5.5F, 75 cm; Abbott, Ladakis, Athens, Greece) was inserted into the right atrium. Intravascular catheters were zeroed to ambient pressure at the phlebostatic axis, and measurements initiated after the systems’ dynamic response was confirmed with fast-flush tests. These allowed the recording of systolic (SAP), diastolic (DAP), and mean (MAP) arterial pressure, and CO, SVR, and right atrial pressure (P_RA_). Arterial blood gases were measured on a blood gas analyzer (IRMA SL Blood Analysis System, Part 436301; Diametrics Medical Inc., Roseville, MN, USA). Baseline data were collected after allowing each animal to stabilize for 30 min.

#### 2.2.5. Preparation of Bacterial Suspensions

We used bacterial suspensions in normal saline with a concentration of approximately 1 × 10^8^ cfu·mL^−1^ and therefore 0.5 McFarland turbidity [9]. The strains (lipopolysaccharide *Escherichia coli* (*E. coli*) ATCC 25922) were derived from the Microbiology Laboratory of the Aretaieion University Hospital in Athens, Greece, and stored at −70 °C in 50% glycerol solution. Each vial contained 5 × 10^8^ cfu·mL^−1^ bacteria in logarithmic phase. Two days prior to the experimental procedure, the vials were allowed to defrost at room temperature and then cultured in blood agar plates. They were incubated at 37 °C for 14 h and then recultured every 14 h. At the middle of the logarithmic phase, the colonies were skimmed from the surface and suspended in 12.5 mL of sterile normal saline that was equally divided into four tubes (3.125 mL each). The 12.5 mL were removed from a sterile normal saline bottle of 100 mL. In each tube, we created a bacterial suspension with a turbidity of 4 McFarland. Then, the suspensions were reinfused back in the 100 mL bottle of normal saline. After vigorous shaking (vortex) we removed 3 mL from the 100 mL and counted the turbidity. If it was 0.5 McFarland, the suspension was accepted. The turbidity was measured with a spectrophotometer at a wavelength of 580 nm (Densicheck Plus Biomerieux). The suspensions were stored at 4 °C for 6–8 h and were left at room temperature 30 min prior to the infusion.

#### 2.2.6. Experimental Procedure

**Figure 1:**
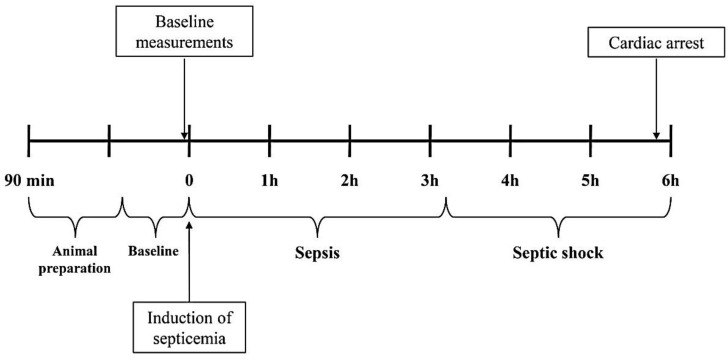
Experimental protocol outline.

After baseline data were collected, septicemia was induced by an intravenous infusion of a bolus of 20 mL of bacterial suspension over two minutes, followed by a continuous infusion (1 mL·kg^−1^·h^−1^; 1 mL = 10^8^ cfu) during the rest of the experiment (Figure 1) [9]. Hemodynamic measurements were obtained every one hour after inoculation and sepsis was documented by the presence of systemic manifestations. The definitions of sepsis and septic shock were based on the 2012 Surviving Sepsis Campaign Guidelines, and septic shock was defined as sepsis-induced hypotension persisting despite adequate fluid resuscitation [23].

Fluid challenges of 10 mL·kg^−1^ isotonic sodium chloride were started with the diagnosis of sepsis-induced arterial hypotension and/or tissue hypoperfusion (lactate > 1 mmol·L^−1^) [23]. Particular attention was paid to infuse the fluid challenges over 20–30 min and not faster in order to prevent an artificial stress response [9]. Norepinephrine infusion of 0.01–3 μg·kg^−1^·min^−1^ targeting a MAP of 65 mmHg was started after three consecutive fluid challenges without improvement in MAP. When MAP ≥ 65 mmHg, septic shock was confirmed and norepinephrine infusion was discontinued [9,23]. No other fluids, vasopressors, or inotropes were used, and no other adjustments were performed despite further deterioration, and all animals were left untreated until cardiac arrest occurred.

#### 2.2.7. Calculation of Baseline Mean Circulatory Filling Pressure Analogue and Related Variables

Mean circulatory filling pressure analog (Pmca) was calculated from running hemodynamic data to assess the effective circulating volume and the driving pressure for venous return. The methods of the Pmca algorithm have been described in detail before [24,25,26,27,28]. Briefly, based on a Guytonian model of the systemic circulation [CO = VR = (Pmcf − P_RA_)/R_VR_], an analogue of Pmcf can be derived using the mathematical model Pmca = (*a* × P_RA_) + (*b* × MAP) + (*c* × CO), where P_RA_ is right atrial pressure and R_VR_ is resistance to venous return [29,30]. In this formula, *a* and *b* are dimensionless constants (*a* + *b* = 1). Assuming a veno-arterial compliance ratio of 24:1, *a* = 0.96 and *b* = 0.04, reflecting the contribution of venous and arterial compartments, and *c* resembles arteriovenous resistance and is based on a formula including age, height, and weight [27,30,31]:

(2) $$c=\frac{0.038\;\left(94.17+0.193\times \mathrm{age}\right)}{4.5\;\left(0.99^{\mathrm{age}-15}\right)\;0.007184\;\cdot \;\left({\mathrm{height}}^{0.725}\right)\;\left({\mathrm{weight}}^{0.425}\right)}\;$$

In addition, the following variables were determined: (1) pressure gradient for venous return (PG_VR_) was defined as the pressure difference between Pmca and P_RA_ [PG_VR_ = Pmca − P_RA_]; (2) resistance to venous return was defined as the ratio of the pressure difference between Pmca and P_RA_ and CO [R_VR_ = (Pmca − P_RA_)/CO], a formula that is used to describe venous return during transient states of imbalances (Pmca is the average pressure in the systemic circulation, and R_VR_ is the resistance encountered by the heart) [32,33]; and (3) efficiency of the heart (Eh) was defined as the ratio of the pressure difference between Pmca and P_RA_ and Pmca [Eh = (Pmca – P_RA_)/Pmca]. This equation was proposed for the measurement of heart performance. During the cardiac stop ejection, P_RA_ is equal to the Pmca, and Eh approaches zero [27,34].

#### 2.2.8. Analysis of the Dynamic Changes in Stressed Volume during Progression of Septic Shock

So as to assess *Vs* during septic shock, we used our extrapolation model in swine of the same age, weight, TBV, and baseline hemodynamics. The baseline *Vs* value and the hourly MAP values during progression of hyperdynamic septic shock were separately determined on a line plot. Using extrapolation lines and linear regression of MAP − *Vs* relationship, we estimated the hourly decrease in *Vs* considering that the total volume status was unchanged.

#### 2.2.9. Calculation of Mean Circulatory Filling Pressure during Cardiac Arrest

Significant changes in vasomotor tone occur after the onset of cardiac arrest. The arterial pressure falls and the venous pressure rises until they almost reach equilibrium [35,36]. Thus, the measurement of Pmcf must be made within the first few seconds after arrest [35,37]. However, the hypotension-induced baroreflex withdrawal maintains an antegrade and pulmonary blood flow that may continue for more than 30–60 s [37]. As Pmcf may vary among individuals, the maximum flow could be better assessed if the time of arrest is more than 20 s [14,38]. Therefore, we initially measured Pmcf using the equilibrium mean P_RA_ between 5 and 7.5 s after the onset of cardiac arrest, before the reflex response had significantly altered the measured plateau pressure [36,39,40]. Then, we continued measuring Pmcf every 10 s until 1 min post-cardiac arrest, provided that the measured plateau pressure was not significantly altered. In this study, Pmcf was measured at six time points (5–7.5 s, 15–17.5 s, 25–27.5 s, 35–37.5 s, 45–47.5 s, and 55–57.5 s post-cardiac arrest).

As arteries are much less compliant than veins, transfer of the remaining arterial volume sufficient to equalize pressures throughout the vasculature could not significantly increase Pmcf or affect measurements in our study [39]. In this context, a plateau was considered adequate to allow accurate measurement if mean P_RA_ rose by less than one mmHg over the period from 5 to 7.5 s after the onset of cardiac arrest [39]. In the present study, all animals had adequate plateau and were included for further analysis.

#### 2.2.10. Statistical Analysis

Statistical analysis was performed using R v4.1. Pearson’s method was used to correlate hemodynamic measurements with Pmca at baseline. Repeated-measures ANOVA was used to assess differences between groups. Linear mixed effects (LME) models were used when needed to assess coefficients additionally to *p*-values. The different subjects (swine) were included as random factor. *p*-values less than 0.05 were deemed significant.

## 3. Results

### 3.1. Progression of Sepsis and Septic Shock

Sepsis progressively evolved with time, and hyperdynamic septic shock was evident after the second hour from induction of septicemia. The progression of sepsis had a significant effect on hemodynamic (Table 1) and metabolic variables (Table S1).

### 3.2. Dynamic Changes in Stressed Volume during Progression of Septic Shock

**Figure 2:**
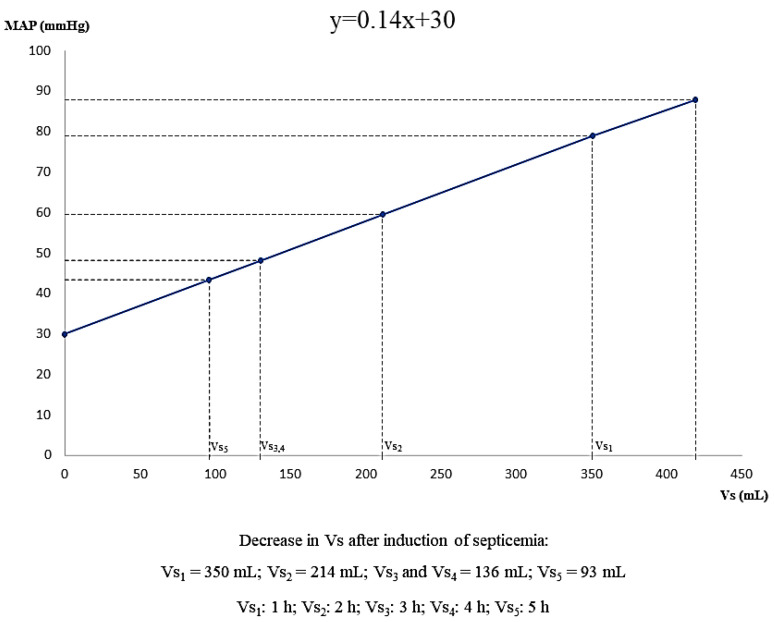
Changes in stressed volume during progression of hyperdynamic septic shock. Cardiac arrest (MAP = 30 mmHg) occurs when Vs = 0. MAP, mean arterial pressure; Vs, stressed volume.

**Figure 3:**
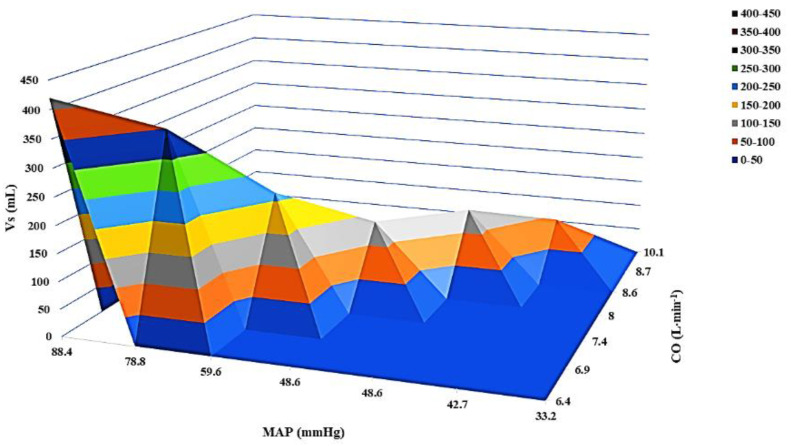
Three-dimensional surface plot showing the functional relationship between stressed volume, mean arterial pressure, and cardiac output during progression of hyperdynamic septic shock. The decrease in stressed volume was the result of progressive vasodilation and was not affected by changes in cardiac output, which increased in an effort to maintain tissue perfusion during progression of shock. Vs, stressed volume; MAP, mean arterial pressure; CO, cardiac output.

The dynamic changes in *Vs* during progression of septic shock are depicted in Figure 2. A 7% decrease in *Vs* was observed for each mmHg decrease in MAP during progression of sepsis and septic shock (Figure 3).

### 3.3. Changes in Mean Circulatory Filling Pressure Analogue and Other Determinants of Venous Return during Septic Shock

Mean circulatory filling pressure analogue decreased with time (*p* < 0.001), along with P_RA_ (*p* < 0.001) and R_VR_ (*p* < 0.001). The PG_VR_ also decreased, but the difference between time points was not statistically significant (*p* = 0.934). In addition, a statistically significant decrease in Eh was observed with time (*p* < 0.001).

### 3.4. Effects of Fluid Challenges and Noradrenaline on Determinants of Venous Return

**Figure 4:**
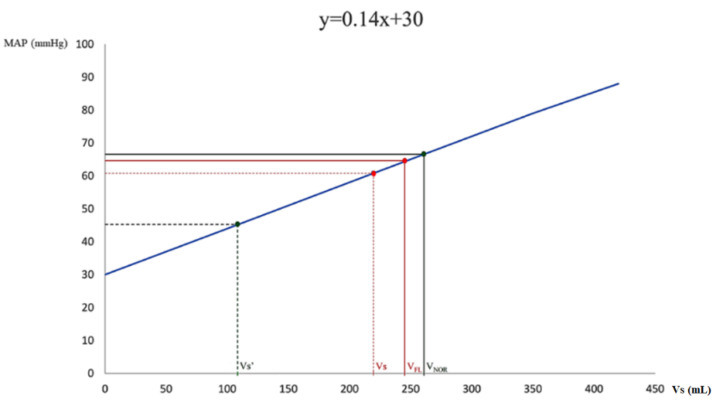
Effect of fluid challenge and noradrenaline on stressed volume. After infusion of 50 mL of isotonic sodium chloride, MAP increased from 61 mmHg to 64 mmHg and Vs increased from 221 mL to 243 mL (V_FL_). After noradrenaline infusion, MAP increased from 45 mmHg to 66 mmHg and Vs increased from 107 mL (Vs’) to 257 mL (V_NOR_). MAP, mean arterial pressure; Vs, stressed volume before fluid infusion; V_FL_, stressed volume after fluid infusion; Vs’, stressed volume before noradrenaline infusion; V_NOR_, stressed volume after noradrenaline infusion.

In total, 30 mL·kg^−1^ were administered within the first three hours from diagnosis of septic shock. The infusion of the first 50 mL of isotonic sodium chloride increased MAP from 61 mmHg to 64 mmHg (5%) and *Vs* from 221 mL to 243 mL (10%). However, neither this nor the subsequent amount of isotonic sodium chloride significantly affected hemodynamics, implying an increase in *Vu* and *Vr* (Table 2, Figure 4).

On the contrary, noradrenaline increased *Vs* from 107 mL to 257 mL (140%) and MAP from 45 mmHg to 66 mmHg (47%). In addition, most systemic hemodynamic variables and determinants of venous return significantly improved after the onset of noradrenaline infusion (Table 3, Figure 4).

### 3.5. Measurement of Mean Circulatory Filling Pressure after Cardiac Arrest

Post-cardiac arrest Pmcf was 14.75 ± 1.5 mmHg. The change in Pmcf during the first minute after cardiac arrest is depicted in Table S2. Baseline Pmca and post-cardiac arrest Pmcf did not differ significantly (14.3 ± 1.23 mmHg vs. 14.75 ± 1.5 mmHg, *p* = 0.24), but the difference between pre-arrest Pmca and post-cardiac arrest Pmcf was statistically significant (9.5 ± 0.57 mmHg vs. 14.75 ± 1.5 mmHg, *p* < 0.001).

## 4. Discussion

The aim of this experimental study was to investigate the dynamic changes in *Vs* and other determinants of venous return during progression and resuscitation of hyperdynamic septic shock in a swine model that closely resembles human hemodynamics. The main findings of the present analysis are: (1) the baseline *Vs* was estimated at 420 mL and decreased by 7% for each mmHg decrease in MAP during progression of septic shock; (2) we revealed a new physiological volume existing at Ptm ≈ 0, the *Vr*, which has important physiological significance and cannot be mobilized without the use of an external vasopressor or without decreasing arterial and/or venous resistance; (3) during septic shock, Pmca, P_RA_, R_VR_, and Eh significantly decreased with time, while PG_VR_ also decreased but did not reach statistical significance; (4) fluid challenges (in total 30 mL·kg^−1^) did not improve systemic parameters or determinants of venous return, while the infusion of noradrenaline significantly improved hemodynamics except for CO, Eh, and R_VR_; and (5) post-cardiac arrest Pmcf did not differ significantly from baseline Pmca, but the difference between pre-arrest Pmca and post-cardiac arrest Pmcf was statistically significant. The present study investigated for the first time the dynamic changes in intravascular volumes and venous return during progression of sepsis to hyperdynamic septic shock and cardiac arrest, providing novel insights into the evolution of cardiovascular dynamics during the condition.

### 4.1. Estimation and Dynamic Changes in Stressed Volume

The evidence on *Vs* estimation in healthy state and sepsis is limited. Ogilvie et al. reported mean *Vs* values of 812 mL (43% of TBV), 952 mL (50% of TBV), and 1148 mL (60% of TBV) for three different ways of inducing circulatory arrest [37]. A model-based computation method of *Vs* from a preload reduction maneuver reported an average *Vs* of 486 mL (22.4% of TBV) in swine [41]. Studies in dogs using the capacity vessel pressure–volume relationship demonstrated *Vs* ranging between 322–653 mL (15–45% of TBV) [42,43,44]. In humans, *Vs* was determined by extrapolating the mean systemic filling pressure (Pmsf, i.e., Pmcf excluding the cardiopulmonary compartment)–volume curve to zero pressure intercept after inspiratory holds and arm stop-flow maneuvers and was estimated to be 1265 mL (≈30% of the predicted TBV) [45]. In another study with postoperative cardiac surgery patients, *Vs* was estimated with inspiratory hold maneuvers at 1677 mL (26% of TBV) [46]. The differences in *Vs* can be explained by the physiological characteristics of species and the method used for its estimation. 

In the present experimental study, *Vs* was estimated at 420 mL and had decreased by 17% after 60 min from the onset of sepsis (no fluid challenges up to this time point), and by 50% after 120 min from the onset of sepsis (100 mL of isotonic sodium chloride had been infused but did not affect *Vs*). Murphy et al. used a three-chambered cardiovascular system model to identify *Vs* in swine and reported that it decreased by 29% after 30–40 min from the infusion of *E. coli* endotoxin [47]. However, 500 mL of saline solution had been administered before endotoxin infusion. Additionally, in a canine model of *E. coli* endotoxin shock, Uemura et al. reported a decrease in *Vs* of 50% after the end of 60 min endotoxin infusion [48]. In either case, it is important to remember that *Vs* and *Vu* are virtual values, not separated, and they change their names and function depending on Ptm at every moment [11]. Nevertheless, the aforementioned data suggest that research on the dynamic changes in *Vs* may lead to distinct shock phenotypes requiring distinct hemodynamic management. Considering the close resemblance between the Landrace–Large White swine and human hemodynamics, this species seems suitable for studying venous return and its determinants in steady and shock states [9,15,49,50].

### 4.2. Conceptual Approach and Characteristics of Rest Volume

One of the most significant findings to emerge from this study is the identification of *Vr* as the volume that cannot be mobilized/converted without the use of an external vasopressor or without decreasing arterial and/or venous resistance, e.g., by decreasing the dose of pure α-adrenergic agonists, such as phenylephrine. The utilization of *Vr* in research and clinical practice is extremely intriguing and helpful. Brengelmann has proposed the same term for the volume (*Vu*) beyond which further addition (in volume) would result in stretching of the vessel walls (distending volume or *Vs*) [51]. However, our analyses show that *Vr* is different from *Vu*, although they both exist at Ptm ≈ 0. In normal conditions, *Vu* can be mobilized, if required, but *Vr* cannot be without external intervention. In particular, *Vr* seems to have dual main functions at the steady state, i.e., to prevent an increase in venous resistance and maintain critical closing pressure, which is the pressure below which small vessels collapse and effective capillary blood flow ceases. As critical closing pressure is related to vascular tone, the *Vr* exerts the peripheral venous pressure required to sustain a vasomotor reflex, resulting in the maintenance of critical closing pressure [52,53]. Indeed, there is evidence showing that profound arterial hypotension during prolonged septic shock may be associated with a drastic increase in venous resistance, especially within the distal part of the splanchnic vasculature [11,54,55]. The aforementioned characteristics of *Vr* mandate that it should not be iatrogenically deranged or should be only minimally affected, even in patients with shock. A severe derangement of *Vr* could explain the devastating effects of exogenous adrenergic agonists, especially when administered in hypovolemic individuals and/or at high doses. 

In our animals, the evolving vasoplegia decreased *Vs* until cardiac arrest occurred (*Vs* = 0 mL, *Vu* = 860 mL, *Vr* = 540 mL). In severe septic shock with low *Vs*, the use of exogenous vasopressors may not be sufficient to completely convert the increased amount of *Vu* (baseline *Vu* plus the converted part of *Vs*) to *Vs*, implying an increase in *Vr* (baseline *Vr* plus part of *Vu* that is not converted to *Vs*) and thus a lower *Vc*. In such a case, increasing vasopressor doses will result in arterial vasoconstriction, increased exit resistance from the arterial compartment, and decreased capillary perfusion [11,56]. In clinical practice, this may be the appropriate time along the pathophysiologic continuum of sepsis/septic shock at which fluid infusion will improve *Vs*, CO, and tissue perfusion. 

Based on the aforementioned characteristics of *Vr*, a drug that stimulates both the α- and β-adrenergic receptors is expected to more effectively maintain systemic hemodynamics than one that activates either α- or β-adrenergic receptors [17]. Indeed, administration of norepinephrine causes arterial and venous constriction and dilatation of the splanchnic vasculature (decreasing splanchnic sequestration at low to moderate doses), which enhance the conversion of *Vu* to *Vs* and facilitate flow through the splanchnic system [11,57], and therefore can improve venous return in patients with septic shock [58].

### 4.3. Dynamic Changes in Mean Circulatory Filling Pressure and Other Determinants of Venous Return

Accurate data on Pmcf in septic patients are also scarce. A meta-analysis investigating the effects of vasopressor-induced hemodynamic changes in adults with shock reported that vasopressor infusion increased Pmsf analogue (Pmsa) from 16 ± 3.3 mmHg to 18 ± 3.4 mmHg, but had variable effects on central venous pressure, Eh, and CO [59]. Guarracino et al. estimated Pmsa in septic shock patients at admission and after resuscitation with fluid and norepinephrine at 13.0 ± 1.4 mmHg and 15.2 ± 1.8 mmHg, respectively, with a PG_VR_ of 6.2 ± 0.8 mmHg [60]. In both Guarracino’s study and our own, fluid resuscitation probably caused hemodilution that decreased and/or prevented an increase in R_VR_ [61,62,63,64,65]. In another study using inspiratory hold maneuvers in septic patients, Pmsf was found to be 26–33 mmHg, depending on the rate of norepinephrine infusion [57]. In the latter study, however, inspiratory holds may have overestimated zero-flow measurements [33]. Of note, Lee et al. investigated the hemodynamic changes in splenectomized dogs after *E. coli* endotoxin infusion and reported an increase in CO concomitantly with a decrease in MAP and Pmsa; however, volume loading (20 mL·kg^−1^) significantly increased Pmsa above baseline values [31]. The improvement in Pmsa can be explained by the pre-endotoxin splenectomy, which prevents volume loss in canine models [65,66]. In the present study, only the first 50 mL of isotonic sodium chloride had a slight effect on MAP, CO, Pmca, and PG_VR_ (R_VR_ and Eh did not change), but neither these nor the total amount of administered fluids (30 mL·kg^−1^) significantly improved hemodynamics. In addition, post-cardiac arrest Pmcf was 14.75 ± 1.5 mmHg in our animals, which was similar to their baseline Pmca, but significantly higher than the Pmca value before the onset of cardiac arrest, implying an increase in *Vu* and *Vr*. In humans, Pmcf measured one minute after death from septic shock was 12.7 ± 5.7 mmHg [67], which is similar to our post-cardiac arrest value. Despite the reported inadequacies in calculating Pmca [37], our findings support its use as a functional hemodynamic monitoring variable to track changes in Pmcf over time, coupling it with other functional hemodynamic parameters in the normal state and septic shock [31,68]. Most especially, the difference between the pre-arrest Pmca and post-cardiac arrest (equilibrium) Pmcf in the present study further strengthens the importance of *Vr* and its characteristics in the healthy state and disease state, as previously discussed in this section.

### 4.4. Clinical Implications

Although the clinical and pathophysiological understanding of septic shock has progressed in the previous decades, many questions still exist. Fluid resuscitation in septic shock is an effective intervention to increase venous return; however, timely fluid resuscitation is critical, and many patients do not respond to treatment [69,70,71]. Administration of fluids is based on the available static and dynamic methods, yet it may also result in overtreatment and organ injury. On the other hand, vasopressor administration can improve systemic hemodynamics, but may not always improve tissue perfusion and may result in adverse effects as well.

The present study revealed the hourly decrease in *Vs* during hyperdynamic septic shock, which increases our understanding of sepsis-induced vasoplegia. As the currently available methods for assessing fluid responsiveness have limitations [72,73,74,75], the use of *Vs* may further support the assessment of the procedure in patients with septic shock. Moreover, our findings can aid in the decision to start vasopressor support according to the decrease in vasomotor tone, a common characteristic of sepsis-related hypotension. Assessment of *Vs* can be also helpful in starting vasopressors simultaneously with fluids or following a very limited fluid resuscitation, which can improve Pmcf/Pmca, venous return, and CO, and decrease net fluid balance, incidence of complications, and mortality [76,77,78,79,80].

In addition, our analysis identified a new circulatory volume, the *Vr*. This volume cannot be mobilized/converted without the use of an external vasopressor or without decreasing arterial and/or venous resistance. The *Vr* seems to have a dual function, i.e., to prevent an increase in venous resistance and maintain critical closing pressure. These findings suggest that fluid management and administration of vasopressors in patients with shock should be considered only if they do not affect or minimally affect the *Vr*. The *Vr* seems extremely important for maintaining hemodynamic homeostasis both in the steady state and disease state.

The present study provides a deeper physiological understanding of hyperdynamic septic shock and new information on how to optimize fluid administration and the use of vasoactive drugs within an individualized treatment strategy. Furthermore, our findings may help in identifying novel phenotypes of septic shock patients.

### 4.5. Strengths and Limitations

The major strength of this experimental study was the resemblance of the hemodynamic and biochemical/metabolic changes during hyperdynamic septic shock between Landrace–Large White swine and humans [9,81]. We acknowledge that this experiment was performed on 10 healthy normovolemic swine and that the use of anesthetics may have affected their response to stress. Nevertheless, the hemodynamic changes during the progression of septic shock were robust. In addition, the present post hoc analysis included only female Landrace–Large White piglets. Furthermore, we did not address the effect of pulsatility on Pmca. However, the oscillations in P_RA_ during the cardiac cycle and vascular buffering minimize this effect [32,33].

## 5. Conclusions

The baseline *Vs* was estimated at 420 mL and decreased by 7% for each mmHg decrease in MAP during progression of hyperdynamic septic shock. Significant changes were also observed in other determinants of venous return. A new physiological intravascular volume existing at Ptm ≈ 0 was identified, termed as *Vr*, which cannot be mobilized/converted without vasopressor support or without decreasing arterial and/or venous resistance.

## Figures and Tables

**Table 1:** Hemodynamic changes in animals during progression of sepsis and septic shock.

	Baseline	1 h	2 h	3 h	4 h	5 h	6 h	*p*-Value
Heart rate (beat·min^−1^)	127.2 (14.23)	137.4 (12.19)	137 (19.09)	134.6 (18.63)	142.7 (18.03)	123.5 (14.94)	129.1 (15.56)	0.135
MAP (mmHg)	88.4 (20.94)	78.8 (20.35)	59.6 (13.50)	48.6 (13.81)	48.6 (15.94)	42.7 (12.26)	33.2 (3.36)	<0.001
CO (L·min^−1^)	6.4 (0.34)	6.9 (0.22)	7.4 (0.25)	8 (0.11)	8.6 (0.19)	8.7 (0.41)	10.1 (0.53)	<0.001
SVR (dynes·sec·cm^−5^)	1012.7 (61.24)	827.5 (42.79)	585.3 (18.06)	443.4 (11.99)	416.2 (14.16)	346.2 (16.98)	244.6 (17.78)	<0.001
P_RA_ (mmHg)	7.3 (1.16)	6.6 (0.84)	5.5 (0.71)	4.1 (0.74)	4 (0.67)	4.9 (0.32)	2.4 (0.52)	<0.001
Pmca (mmHg)	14.3 (1.23)	13.5 (0.85)	11.9 (0.74)	10.5 (0.71)	10.8 (0.64)	11.5 (0.38)	9.5 (0.57)	<0.001
PG_VR_ (mmHg)	6.9 (0.16)	6.9 (0.11)	6.4 (0.18)	6.4 (0.08)	6.8 (0.12)	6.6 (0.24)	7.1 (0.3)	0.934
R_VR_ (mmHg·min·L^−1^)	1.1 (0.03)	1 (0.02)	0.87 (0.01)	0.8 (0.01)	0.79 (0.01)	0.75 (0.01)	0.7 (0.01)	<0.001
Eh	0.49 (0.04)	0.52 (0.03)	0.54 (0.03)	0.61 (0.04)	0.63 (0.04)	0.57 (0.02)	0.75 (0.04)	<0.001
Vs (mL)	420	350	214	136	136	93	≈0	<0.001

Values are expressed as mean (SD). MAP, mean arterial pressure; CO, cardiac output; SVR, systemic vascular resistance; P_RA_, right atrial pressure; Pmca, mean circulatory filling pressure analog; PG_VR_, pressure gradient for venous return; R_VR_, resistance to venous return; Eh, efficiency of the heart.

**Table 2:** Effect of fluid challenges on hemodynamic variables.

	2 h (100 mL)		3 h (300 mL)		4 h (200 mL)		
	Before	After	Before	After	Before	After	*p*-Value
Heart rate (beat·min^−1^)	140 (15)	126 (7)	138 (4)	137 (6)	148 (12)	148 (9)	1
MAP (mmHg)	61 (11)	64 (6)	46 (5)	46 (6)	45 (7)	45 (4)	1
CO (L·min^−1^)	7.1 (2)	7.3 (2)	7.9 (2)	8 (2)	8.5 (2)	8.6 (2)	0.79
SVR (dynes·sec·cm^−5^)	629 (14)	642 (8)	424 (16)	420 (11)	386 (24)	381 (17)	0.98
P_RA_ (mmHg)	5.2 (0.2)	5.4 (0.5)	4.1 (0.3)	4 (0.2)	4 (0.4)	4 (0.5)	1
Pmca (mmHg)	11.6 (0.4)	12 (0.3)	10.4 (0.8)	10.3 (0.2)	10.6 (0.3)	10.6 (0.3)	1
PG_VR_ (mmHg)	6.4 (0.5)	6.6 (0.2)	6.3 (0.2)	6.3 (0.3)	6.6 (0.3)	6.6 (0.1)	1
R_VR_ (mmHg·min·L^−1^)	0.9 (0.1)	0.9 (0.2)	0.8 (0.2)	0.8 (0.3)	0.8 (0.2)	0.8 (0.2)	1
Eh	0.55 (0.02)	0.55 (0.03)	0.61 (0.01)	0.61 (0.01)	0.62 (0.01)	0.62 (0.01)	1
Vs (mL)	221	243	119	119	119	119	0.962

Values are expressed as mean (SD). MAP, mean arterial pressure; CO, cardiac output; SVR, systemic vascular resistance; P_RA_, right atrial pressure; Pmca, mean circulatory filling pressure analog; PG_VR_, pressure gradient for venous return; R_VR_, resistance to venous return; Eh, efficiency of the heart.

**Table 3:** Effect of noradrenaline on hemodynamic variables.

	Before	After	*p*-Value
Heart rate (beat·min^−1^)	147 (8)	119 (9)	<0.001
MAP (mmHg)	45 (5)	66 (1)	<0.001
CO (L·min^−1^)	8 (2)	8.6 (2)	0.510
SVR (dynes·sec·cm^−5^)	410 (11)	572 (9)	<0.001
P_RA_ (mmHg)	4 (0.2)	4.5 (0.1)	<0.001
Pmca (mmHg)	10.3 (0.3)	11.9 (0.2)	<0.001
PG_VR_ (mmHg)	6.3 (0.1)	7.4 (0.1)	<0.001
R_VR_ (mmHg·min·L^−1^)	0.8 (0.2)	0.9 (0.1)	0.174
Eh	0.61 (0.01)	0.62 (0.01)	0.826
Vs (mL)	107	257	<0.001

Values are expressed as mean (SD). MAP, mean arterial pressure; CO, cardiac output; SVR, systemic vascular resistance; P_RA_, right atrial pressure; Pmca, mean circulatory filling pressure analog; PG_VR_, pressure gradient for venous return; R_VR_, resistance to venous return; Eh, efficiency of the heart.

## Data Availability

Data can be made available upon request after publication through a collaborative process. Researchers should provide a methodically sound proposal with specific objectives in an approval proposal. Please contact the corresponding author for additional information.

## Supplementary Materials

The following supporting information can be downloaded at: https://www.mdpi.com/article/10.3390/jpm12050724/s1. Figure S1: Total blood volume in a 20-kg swine; Table S1: Metabolic changes in animals during progression of sepsis and septic shock; Table S2: Mean circulatory filling pressure after cardiac arrest.
