# Supplementary material for: Assessment of Dynamic Changes in Stressed Volume and Venous Return during Hyperdynamic Septic Shock
Source: J Pers Med. 2022 Apr 29;12(5):724. doi: 10.3390/jpm12050724 (PMC9146182; doi:10.3390/jpm12050724)
Supplement: Supplementary file 1 [file jpm-12-00724-s001.zip › jpm-1686892-supplementary/Figure S1.pdf]

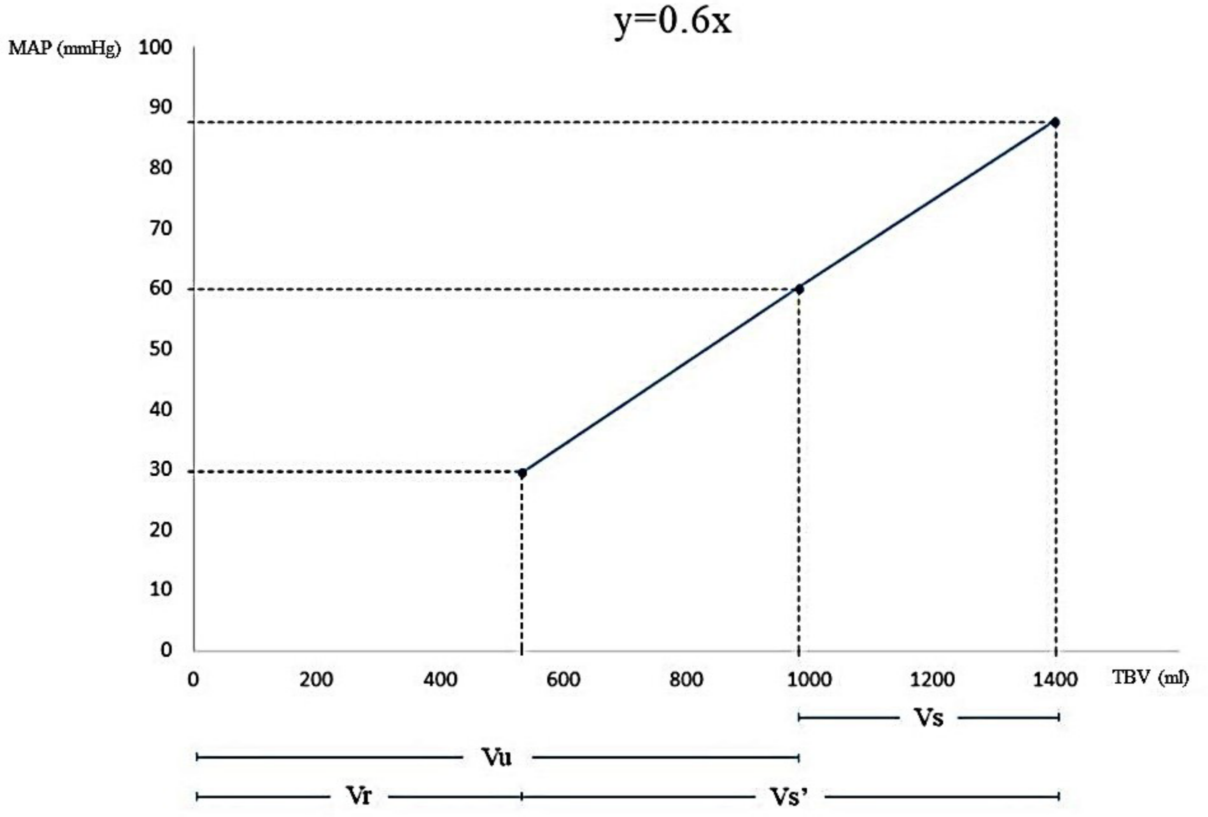

Figure S1. Total blood volume in a 20 kg-swine.

Vs, stressed volume; Vu, unstressed volume; Vr, rest volume Vs′, volume that can mobilized for the splanchnic and other compliant veins. Data are derived from reference 15.
