# Supplementary material for: Assessment of Dynamic Changes in Stressed Volume and Venous Return during Hyperdynamic Septic Shock
Source: J Pers Med. 2022 Apr 29;12(5):724. doi: 10.3390/jpm12050724 (PMC9146182; doi:10.3390/jpm12050724)
Supplement: Supplementary file 1 [file jpm-12-00724-s001.zip › jpm-1686892-supplementary/Table S1.pdf]

Table S1. Metabolic changes of animals during progression of sepsis and septic shock

	Baseline	0	1 h	2 h	3 h	4 h	5 h	6 h	p-value
SpO_2 (%)	94.1 (3.8)	93.7 (4.4)	90.2 (3.6)	89.9 (4.7)	86.3 (5.5)	83.8 (5.4)	83.2 (4.8)	78.5 (3.4)	0.026
$ETCO_2$ (mmHg)	40.2 (4.65)	33.41 (1.14)	32.1 (3.39)	30.4 (2.79)	26.23 (5.01)	24.52 (4.35)	26.53 (2.12)	26.14 (2.82)	0.001
pH	7.44 (0.06)	7.43 (0.08)	7.42 (0.06)	7.28 (0.1)	7.06 (0.2)	7.08 (0.22)	7.04 (0.13)	7.05 (0.17)	0.031
PaO_2 (mmHg)	92.78 (4.76)	93.38 (3.52)	90.82 (2.72)	70.64 (5.84)	69.32 (5.44)	64.1 (3.88)	54.3 (6.39)	49.6 (6.56)	0.001
$PaCO_2$ (mmHg)	39.56 (1.22)	39.64 (1.58)	39.94 (2.37)	44.86 (4.78)	42.46 (5.45)	43.6 (4.36)	45.3 (5.32)	71.62 (7.89)	0.001
PaO_2/FiO_2	441.8 (22.6)	444.66 (16.76)	432.47 (12.95)	336.38 (27.8)	330.1 (25.9)	305.23 (18.47)	258.57 (30.42)	236.19 (31.23)	<0.001
HCO_3 ($mmol{\cdot}L^{-1}$)	27.32 (3.79)	28.7 (1.78)	31.74 (1.47)	30.1 (1.6)	23.72 (1.26)	25.5 (1.78)	20.34 (1.65)	19.1 (1.63)	0.001
EB ($mmol{\cdot}L^{-1}$)	4.98 (3.53)	5.1 (2.23)	3.1 (1.78)	1.92 (1.12)	-1.52 (1.32)	-1.66 (1.80)	-6.7 (1.44)	-11.5 (1.45)	0.001
Lactate ($mmol{\cdot}L^{-1}$)	0.9 (0.02)	0.8 (0.24)	0.9 (0.12)	2.2 (0.3)	2.9 (0.5)	3.5 (0.2)	4.2 (0.4)	5.1 (0.5)	0.03

Values are in mean (SD).

SpO_2 = oxygen saturation of hemoglobin, $ETCO_2$ = end-tidal carbon dioxide, PaO_2 = partial arterial oxygen pressure, $PaCO_2$ = partial arterial carbon dioxide pressure.
