# Supplementary material for: Assessment of Dynamic Changes in Stressed Volume and Venous Return during Hyperdynamic Septic Shock
Source: J Pers Med. 2022 Apr 29;12(5):724. doi: 10.3390/jpm12050724 (PMC9146182; doi:10.3390/jpm12050724)
Supplement: Supplementary file 1 [file jpm-12-00724-s001.zip › jpm-1686892-supplementary/Table S2.pdf]

Table S2. Mean circulatory filling pressure after cardiac arrest

Time after cardiac arrest	Mean (mmHg)	SD (mmHg)
5-7.5 seconds	15.09	1.86
15-17.5 seconds	14.73	1.85
25-27.5 seconds	14.9	1.79
35-37.5 seconds	14.7	1.37
45-47.5 seconds	14.53	1.12
55-57.5 seconds	14.54	0.86

The differences between time points were not statistically significant.
